# Dirofilaria immitis conquering the regions in Slovakia previously endemic for D. repens

**DOI:** 10.1007/s00436-023-07983-4

**Published:** 2023-09-29

**Authors:** Martina Miterpáková, Daniela Valentová, Zuzana Hurníková

**Affiliations:** 1 Institute of Parasitology, Slovak Academy of Sciences, Hlinkova 3, 040 01 Košice, Slovakia; 2 Veterinary and Food Institute, Botanická 15, 842 52 Bratislava, Slovakia

**Keywords:** Dirofilariosis, *Dirofilaria immitis*, Heartworm disease, Endemic Region, Europe

## Abstract

The present study was focused on the current state of *Dirofilaria* species distribution in the territory of the Slovak Danubian Lowland, a region previously identified endemic for *Dirofilaria repens*. For the research, blood samples of 330 dogs tested positive for dirofilariosis using concentration tests or “rapid heartworm tests” were sent by private veterinary practitioners for further DNA analyses and species determination. The results revealed an unquestionable change in the pattern of *Dirofilaria* species distribution with *Dirofilaria immitis*, diagnosed as the agent of mono- and co-infections with *D. repens*, responsible for 60.00% of all cases. The results showed that in the course of the last 5 years, *D. immitis* has spread significantly in Slovakia and has become the dominant causal agent of dirofilariosis in the former endemic areas of *D. repens* distribution, which increases infection risk for both dogs and humans.

## Introduction

*Dirofilaria immitis* and *Dirofilaria repens*, mosquito-transmitted parasitic filarioids, are of great concern in Europe for several decades. Dogs are the main hosts for both species with *D. immitis* causing severe cardiopulmonary “heartworm” disease (HWD) and *D. repens* responsible for the subcutaneous and ocular form of the infection. Both species have a zoonotic character, but in Europe, the majority of human cases of dirofilariosis are associated with *D. repens* (ESDA 2017).

From the epidemiological point of view, the recent data show a stable situation or even a decreasing trend in *D. immitis* prevalence in Western European countries, including previously hyperendemic areas, and a faster spread of *D. repens* in these territories (Genchi and Kramer 2020). Contrary to this, in several regions of Europe, including the Great Hungarian Plain or Apulia region and Linosa island in Southern Italy, *D. immitis* prevalence increased evidently in a short period (Mendoza-Roldan et al. 2020; Fuehrer et al*.* 2021; Brianti et al. 2023). This situation was also observed in Slovakia, Central Europe, where canine dirofilariosis was reported for the first time in 2005 (Svobodová et al. 2005). The area-wide epidemiological investigations that were performed afterward revealed the presence of *D. repens* in high-endemic areas with an overall prevalence of over 30.0% and with the highest positivity recorded among working dogs (Miterpáková et al. 2008, 2010).

During the first decade of epidemiological research on canine dirofilariosis in Slovakia (between 2005 and 2015), *D. immitis* cases were recorded only sporadically (Miterpáková et al. 2016). However, the situation has recently changed significantly and an evident increasing trend in the number of heartworm infections has been observed (Miterpáková et al. 2018, 2020).

The present study aimed to evaluate the current distribution of *Dirofilaria* species in the population of dogs with microfilaremia from the previously identified hyperendemic region.

## Material and methods

### Study area and sample collection

**Fig. 1:**
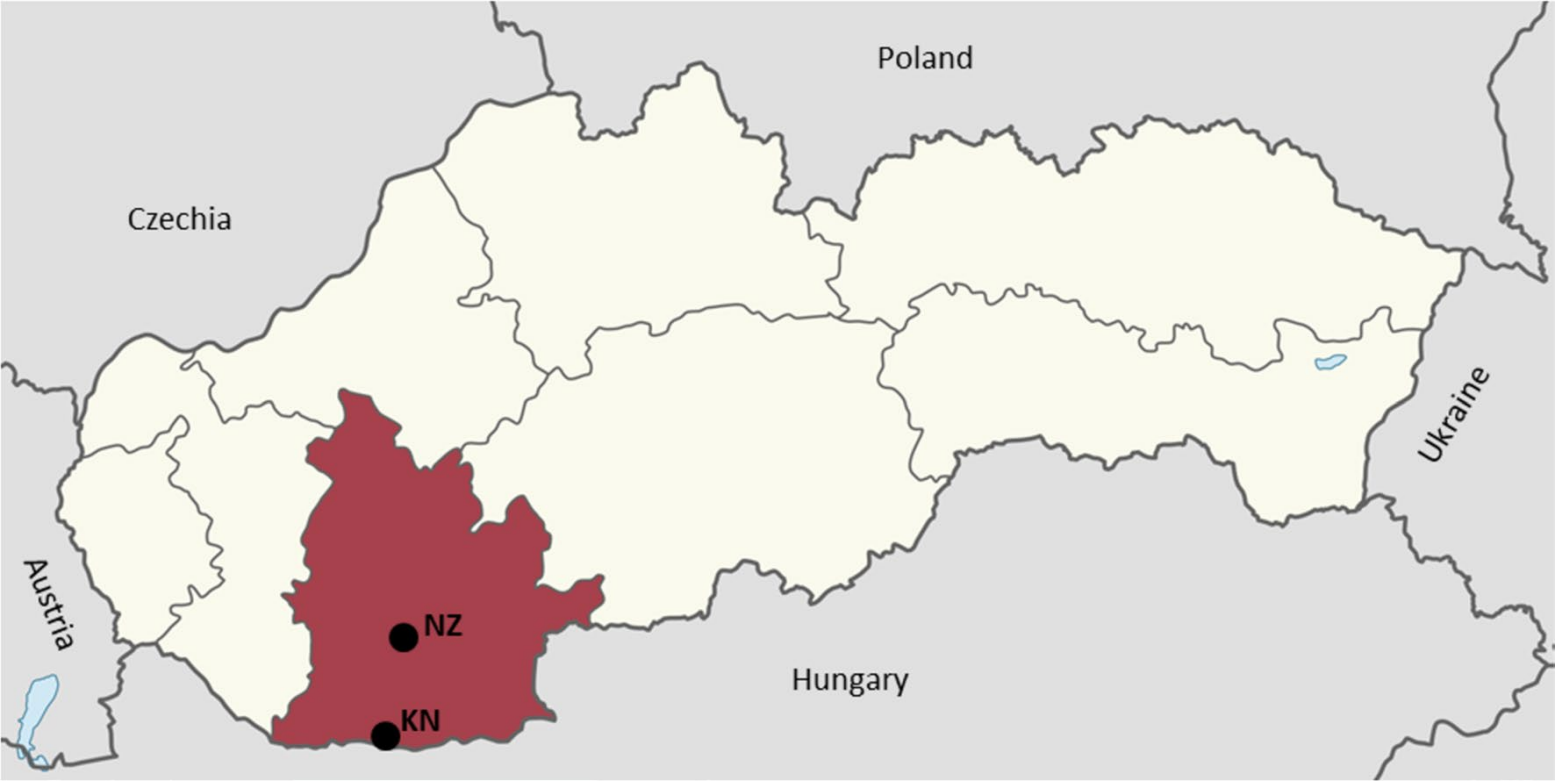
The region of sampling of *Dirofilaria* spp. positive blood (districts Komárno (KN) and Nové Zámky (NZ), Slovakia)

The present study was carried out in a previously identified endemic region of canine subcutaneous dirofilariosis situated in the southwestern part of Slovakia represented by the Slovak Danubian Lowland near the Slovak-Hungarian borders. The majority of the dogs examined came from districts Komárno and Nové Zámky (Fig. 1).

Between 2019 and 2022, private veterinary practitioners residing in the study regions were asked to send blood samples from dogs diagnosed with dirofilariosis to the Institute of Parasitology of the Slovak Academy of Sciences in Košice (IP SAS) and Veterinary and Food Institute in Bratislava (VFI) for *Dirofilaria* species identification and further analyses. All samples were accompanied by questionnaire forms with data concerning age, breed, sex, travel history, locality of residence, and health status.

### Diagnostic procedures

In veterinary practices, *Dirofilaria* spp. infections were diagnosed using fresh blood smear or Knott’s test (Knott 1939) to detect microfilariae or commercial rapid test system based on enzyme immunoassay technique for presence of circulating *D. immitis* antigen (Rapid CHW Ag Test Kit; BioNote, Inc., Republic of Korea or SNAP Heartworm RT Test; IDEXX Laboratories, Inc., Westbrook, ME, USA). All dogs confirmed positive using the heartworm test were subsequently examined also by Knott’s test.

All samples that tested positive for microfilariae and/or *D. immitis* antigen (*n* = 330) were delivered to IP SAS for *Dirofilaria* species confirmation employing DNA analyses. For this purpose, DNA was extracted from 200 μl of blood with DNeasy Blood & Tissue Kit (Qiagen, Hilden, Germany) and afterward tested using a conventional PCR approach which amplifies a 203-bp fragment of the cytochrome c oxidase subunit 1 (*cox*1) gene of *D. immitis* and o 209-bp portion *of D. repens* COI gene according to Rishniw et al. (2006).

## Results

### *Dirofilaria* species occurrence

Within the study, altogether 330 dogs were found to be positive for canine dirofilariosis by private veterinary practitioners. The age of the dogs varied from 1 to 15 years (in 26 individuals the age was not referred to); 144 of them were females and 186 were males. Out of 330 dogs, 170 were kept by private owners, and 160 came from two dog shelters situated in Komárno and Nové Zámky districts.

**Fig. 2:**
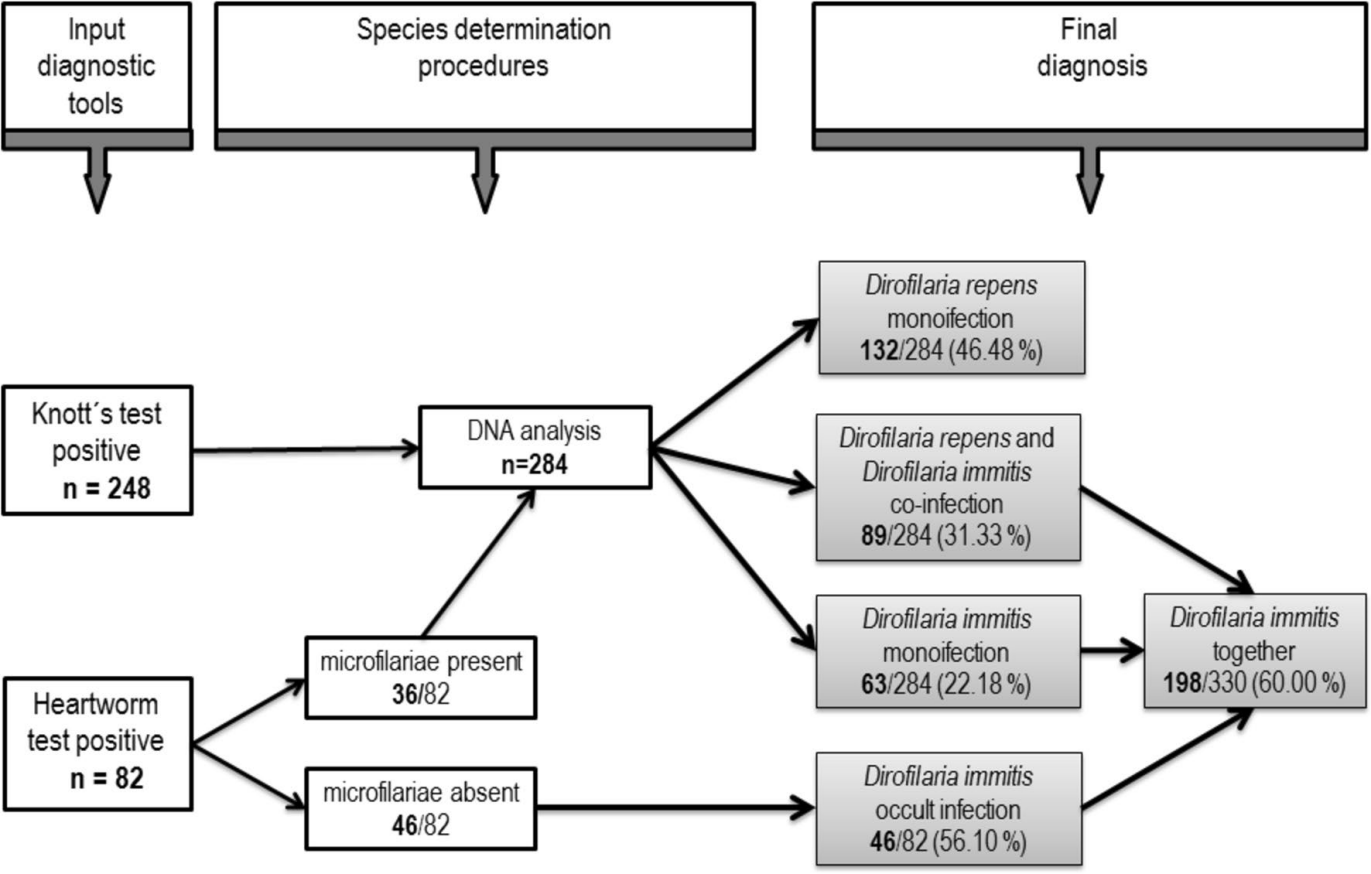
The study flowchart: *Dirofilaria* species determination procedures in 330 dogs tested positive for dirofilariosis

The diagnosis was predetermined based on microfilariae presence (*n* = 284) and/or positive result of the heartworm antigen test (*n* = 82). Thirty-six of 82 *D. immitis*–seropositive dogs revealed also microfilariae in the blood; contrary, the remaining 46 seropositive dogs tested negative using Knott’s test. The diagnostic procedures and their results are illustrated in Figure 2.

DNA analysis of 284 microfilaraemic blood samples confirmed the mono-infection with *D. repens* in 132 dogs (46.48%), *D. immitis* in 63 animals (22.18%), and *D. repens* and *D. immitis* co-infection in 89 individuals (31.33%). In total, heartworm infection, confirmed either by DNA analysis or by serology, was diagnosed in 198 out of 330 dogs examined, which represents 60.00% of *Dirofilaria*-positive dogs included in the study (Figure 2).

Considering dogs from shelters independently, the majority of the animals, 79 of 160, were infected with *D. immitis* (49.38%), in 42 (26.25%) of them co-infection with both dirofilarial species was confirmed, and in 39 (24.38%) the mono-infection with *D. repens* was diagnosed.

### Clinical manifestation

According to questionnaires that accompanied each sample, the changes in health conditions were observed in 82 infected dogs (24.85%). Out of 132 dogs that tested positive for *D. repens* infection, 44 (33.33%) showed clinical signs to some extent, most frequently the presence of subcutaneous nodules (27×), followed by pyometra observed in eight dogs, cachexia in five, and renal failure in four infected animals.

The mixed dirofilarial infection diagnosed in 89 dogs was clinically manifested in 30 individuals (33.71%) with clinical signs including most often chronic cough (15×), tachypnea (7×), and pneumonia (6×), followed by heart murmurs (4×), ascites (3×), hydropericardium syndrome (2×), dermatitis (2×), hepatopathy (1×), pancreatitis (1×), and effusion in the thorax (1×). Several dogs suffered simultaneously from various symptoms.

In eight of 63 (12.70%) dogs with diagnosed *D. immitis* mono-infection, heart failure–related ascites was observed; in three of them, also adult worms were found *post-mortem* in the right heart ventricle and pulmonary arteries. One dog with heartworm infection suffered also from haemolytic anaemia.

## Discussion

The data analysed in the present study show an unquestionable change in the pattern of *Dirofilaria* species distribution in dogs living in the southwestern region of Slovakia over the recent 5 years in a favour of more pathogenic *D. immitis*. The results exposed that the number of dogs with heartworm diseases (HWD) has increased progressively, and *D. immitis* was responsible for more than half of all cases of canine dirofilariosis registered by veterinarians in surveyed districts Komárno and Nové Zámky (Fig. 2). A similar trend was reported in Hungary, neighbouring Slovakia from the south, where *D. immitis* recently became hyperendemic in the Great Hungarian Plain, previously considered endemic only for *D. repens* (Farkas et al. 2020; Széll et al. 2020). Also in Ukraine, bordering both Slovakia and Hungary, despite the shortness of accessible data, the current situation points to an increasing trend of *D. immitis* infections in the dog population. Most information is available from the Kharkiv region lying in northern Ukraine on the borders with Russia where several epidemiological studies were performed recently. In a survey carried out between September 2018 and February 2019, a total of 112 dogs were examined for dirofilariosis. The infection was diagnosed in 24 (21.4%) of them with *D. immitis* confirmed in 14 and *D. repens* in ten individuals (Kryvoruchenko et al. 2019). Additionally, during the winter hunting season in 2019/2020, 27 red foxes from the same region were examined by autopsy, and six of them were found positive for *D. immitis* adult worms localized in the right heart ventricle, pulmonary trunk, and pulmonary arteries (Liulin et al. 2021). On the other hand, in three other countries bordering Slovakia, Austria, Czechia, and Poland, *D. immitis* still occurs only sporadically, mainly as an agent imported from the Mediterranean region (Fuehrer et al. 2021). Interestingly enough, the quite recent epidemiological study performed in parallel on the territory of Czechia and Slovakia, the neighbouring countries with a common social and political background as well as similar veterinary care levels, revealed significant differences in the distribution of *Dirofilaria*-infected dogs with the recorded prevalence of 1.86% and 10.56%, respectively (Miterpáková et al. 2021). The epidemiological situation concerning canine dirofilariosis is changing also in historically endemic regions, e.g. the decreasing trend of HWD cases in the previously hyperendemic area in Northern Italy is accompanied by simultaneous expansion of *D. immitis* towards southern, previously non-endemic regions (Genchi and Kramer 2020; Panarese et al. 2020a). Apart from environmental changes, the reason for this reverse trend of *D. immitis* distribution could be explained by the regular long-term use of chemoprophylaxis treatment in previously endemic areas and its absence in regions where veterinary practitioners are not aware of HWD yet.

The lack of preventive measures and adequate treatment could also be the main reason for high *D. immitis* prevalence in the shelter and street dogs representing a suitable source for the infection spread (Otranto et al. 2017; Panarese et al. 2020a). This fact was backed also by the results of our study seeing that many dogs with diagnosed HWD, caused by *D. imm*itis or co-infection with *D. repens*, came from shelters. From the epidemiology point of view, it is important to note that high numbers of dogs kept in Slovak shelters are exported abroad, most frequently to Austria, Germany, and Switzerland (personal information with shelter managers and veterinarians), which can contribute to the expansion of the infection agents into non-endemic regions.

Considering that in more than one half of *D. immitis* seropositive dogs (46 out of 82; 56.10%), microfilariae were absent in the peripheral blood, it could be suspected that the true prevalence of HWD in Slovakia is underestimated. The high probability of occult *D. immitis* infections, caused by a small number of microfilariae, unisex infestation, testing within the incubation period, or drug-induced sterility of the female worms, has been reported by several authors (e.g. Rawling et al. 1982; Pantchev et al. 2011). One of the most recent studies carried out by Panarese et al. (2020b) in a high-risk geographic area in Southern Italy revealed that the prevalence of canine dirofilariosis varied between 44.4 and 79.6% according to the diagnostic method used. From the practical point of view, the use of Knott’s (or another concentration) test alone may lead to false-negative results; therefore, a diagnostics of canine dirofilariosis in veterinary clinics should, at least in endemic regions, include also a rapid serological test for heartworm antigen detection. Nevertheless, from another point of view, the question regarding the sensitivity and specificity of the serological tests arises. A lot of studies based on various antigen tests detecting heartworm disease have been carried out worldwide with a very different sensitivity unveiled. For instance, several recent comparative studies revealed rather great differences in sensitivity (varied between 76.9 and over 97.0%) and specificity (ranged from 66.7 to 99.5%) of tests routinely used in veterinary praxes in the USA and Europe (Burton et al. 2020; Becker et al. 2022). For that reason, also dogs from our study diagnosed with the occult form of *D. immitis* infection should be correctly defined as “suspected for heartworm disease”. Anyway, the research of occult dirofilariosis needs more attention, especially in currently formed endemic areas where both dirofilarial species circulate.

On the other hand, dogs with microfilariaemia, in particular individuals without clinical manifestation and any adequate treatment, represent the main source for the spread of infection. Veterinarians in endemic regions should therefore take this possibility into account and focus more widely on prophylactic measures.

## Conclusion

The presented study confirmed that Slovakia, endemic for *D. repens* since 2007, became endemic for *D. immitis* in the course of recent 5 years, whereby the endemic regions are identical for both species. From a practical point of view, to reduce the risk of infection and given the zoonotic nature of the parasites, it is essential to continue the epidemiological surveillance of heartworm disease and to emphasise awareness raising among veterinarians and dog breeders.
